# Perceived AI interview design and organizational attractiveness: the roles of social presence, telepresence, and AI literacy

**DOI:** 10.3389/fpsyg.2026.1836040

**Published:** 2026-06-24

**Authors:** Jie Yu, Seon-Joo Kim

**Affiliations:** 1 Department of Educational Consulting, Graduate School, Pukyong National University, Busan, Republic of Korea; 2 College of Future Convergence, Pukyong National University, Busan, Republic of Korea

**Keywords:** AI interview, AI literacy, organizational attractiveness, social presence, telepresence

## Abstract

**Introduction:**

This study examines how candidates' perceptions of AI interview design features influence organizational attractiveness through their anticipated experience of standardized, AI-mediated selection. Applying the stimulus-organism-response (S-O-R) framework, we propose a presence-based model linking three specific design features-human-likeness cues, decision transparency, and feedback informativeness-to social presence and telepresence, which in turn relate to organizational attractiveness as a candidate-side recruitment outcome.

**Methods:**

Survey data were collected from 755 job seekers who evaluated a standardized AI interview scenario. The proposed hypotheses were tested using structural equation modeling (SEM).

**Results:**

Social presence functioned as the primary and more consistent mechanism linking perceived design features to organizational attractiveness, whereas telepresence played a more constrained role. Candidates' self-reported AI literacy strengthened the positive association between telepresence and organizational attractiveness, but did not significantly moderate the relationship between social presence and organizational attractiveness.

**Discussion:**

These findings indicate that candidates' evaluations of AI-mediated recruitment depend not only on formal procedural features but also on the perceived experiential quality of the interaction. By clarifying the presence-based mechanisms underlying candidate reactions, this study extends the literature on technology-mediated selection and offers practical guidance for designing human-centered, candidate-sensitive AI recruitment systems.

## 1 Introduction

Recent advances in digital technologies have transformed human resource management, especially recruitment and selection. Organizations increasingly use AI-enabled tools for résumé screening, candidate assessment, and interviewing (Dima et al., 2024; Jarrahi, 2018; Tursunbayeva et al., 2025). Among these tools, technology-mediated and AI-mediated interviews have become a visible part of contemporary selection practice (Acikgoz et al., 2020; Blacksmith et al., 2016; Lukacik et al., 2022). In such settings, AI systems may evaluate candidates' responses and generate assessments used in selection decisions (Hickman et al., 2022). As these systems become more common, candidates are likely to respond to both interview content and the way the interview system is experienced. The design of the system may therefore shape how candidates make sense of the interview process and, ultimately, how they judge the hiring organization (Goretzko and Finja Israel, 2022). Research on candidate reactions to AI-based selection has focused mainly on fairness, transparency, and trust (Malin et al., 2025; Nørskov et al., 2020; Suen et al., 2019). Recent studies have also examined candidates' fairness perceptions of algorithmic recruitment tools, drawing attention to concerns about limited behavioral control and reduced social connection in automated selection processes (Hilliard et al., 2022). Although this work has generated important insights, it says less about how candidates experience an AI-mediated interview as an interaction in its own right. In highly standardized, technology-mediated settings, candidates may respond to whether the system seems fair and accurate, as well as to whether the interaction feels socially engaging and immersive. This issue is particularly salient in AI-mediated interviews, where direct interpersonal contact is reduced even though the interview remains a highly visible and symbolically important stage of the recruitment process (Nørskov et al., 2020; Suen et al., 2019). Procedural judgments alone may therefore be insufficient to explain candidate responses to AI interviews. Organizational justice and trust in AI are both highly relevant to AI-mediated selection. Justice perspectives explain how candidates evaluate the fairness of procedures, explanations, interpersonal treatment, and outcomes, whereas trust-based perspectives are especially useful for understanding whether candidates view an AI system as reliable, credible, and acceptable. The present study does not treat these perspectives as irrelevant. Rather, it addresses a different theoretical question: how visible design features of an AI-mediated interview shape candidates' experiential states and how these experiences are translated into organizational attractiveness. The S–O–R framework is therefore used as the primary organizing lens because it is well suited to modeling a process in which external design cues operate as stimuli, perceived presence represents the candidate's internal experiential state, and organizational attractiveness reflects the evaluative response. In this sense, organizational justice and trust in AI are treated as adjacent and complementary perspectives rather than as the central theoretical mechanisms of the present model. Because these interviews are encountered through a mediated interface rather than direct interpersonal contact, candidates may form impressions of the organization from the quality of the mediated experience itself (Kojima et al., 2021; Lombard and Ditton, 1997; Parker et al., 1976; Steuer, 1992; Ye et al., 2020). To address this gap, the present study develops a presence-based model of candidate reactions in AI-mediated interviews. Specifically, it examines three perceived interview design features—AI interviewer human-likeness cues, decision transparency, and feedback informativeness—and how they shape social presence and telepresence, relate to organizational attractiveness, and vary with candidates' AI literacy. These design features capture how candidates interpret an AI-mediated interview as an organizational interaction rather than merely a technical assessment setting (Gilliland, 1993; Qiu and Benbasat, 2009; Suen and Hung, 2023). Together, these features reflect three candidate-facing cues that are especially visible in an AI-mediated evaluative encounter: the social character of the interviewer, the intelligibility of the evaluation process, and the communicative value of feedback. Organizational attractiveness is especially relevant here because candidates do not evaluate AI-mediated interviews in isolation. Instead, they may read the interview experience as a signal of how the organization communicates, exercises judgment, and treats candidates during the selection process. Impressions formed in an AI-mediated interview may therefore carry over into evaluations of the organization as a potential employer.

This study contributes to the literature in three ways. It first extends research on applicant reactions by moving beyond a predominantly procedural account of AI-mediated interviews toward an experience-based organizational psychology perspective. The central argument is that candidates evaluate AI interviews through both procedural judgments, such as fairness and transparency, and the quality of the interaction within a technology-mediated selection encounter. The study also advances work on AI-mediated interviews by distinguishing between social presence and telepresence rather than treating mediated experience as a single undifferentiated perception. This distinction clarifies that the two forms of presence play different roles in shaping organizational attractiveness as a recruitment-related outcome. Finally, the study identifies self-reported AI literacy as a boundary condition that shapes when AI-mediated interview experiences are translated into organizational evaluations, thereby linking candidates' perceived ability to understand and evaluate AI systems with their reactions to AI-mediated interviews (Pinski and Benlian, 2024; Tulcanaza-Prieto et al., 2025).

AI-mediated interviews have become increasingly common in contemporary personnel selection (Acikgoz et al., 2020). Broadly, they are interview systems in which algorithmic technologies are used to structure, record, and evaluate candidate responses in support of hiring decisions (Langer et al., 2019). Compared with traditional interviews, this format reduces direct human involvement and changes how candidates experience the selection process. Prior research suggests that reactions to AI-mediated interviews are often less favorable than reactions to conventional human-led interviews, particularly when the process is perceived as impersonal, opaque, or lacking interpersonal responsiveness (Nørskov et al., 2020; Suen et al., 2019). Related work has also shown that AI-mediated or digitally mediated interview formats may heighten uncertainty, limit opportunities for spontaneous self-presentation, and shape candidates' affective responses to the selection process (Köchling et al., 2023; Langer et al., 2017). At the same time, recent studies suggest that candidate responses vary depending on how AI interviews are designed and how their features are interpreted (Min et al., 2024; Ochmann et al., 2024; Suen and Hung, 2023). AI-mediated interviews should therefore be understood as candidate-facing interaction settings in which system design shapes interview experience and organizational evaluations.

Presence offers a useful lens for understanding how candidates experience AI-mediated interviews as mediated organizational encounters rather than as merely technical assessment procedures. In mediated communication research, presence refers to the psychological experience through which a technology-enabled interaction or environment becomes subjectively meaningful to the user (Lombard and Ditton, 1997; Steuer, 1992). This perspective is particularly relevant to AI-mediated interviews because candidates encounter the hiring organization through an interface that asks questions, records responses, communicates evaluation cues, and shapes the felt quality of the interview process. Within this experience, social presence and telepresence capture related but distinct dimensions. Social presence concerns the extent to which the mediated interaction conveys social connection, interpersonal warmth, responsiveness, and a sense of being acknowledged by an interaction partner (Parker et al., 1976; Verhagen et al., 2014). In AI-mediated interviews, such presence does not require the physical involvement of a human interviewer; it may arise when an AI interviewer or interface is perceived as responsive and socially meaningful enough to be experienced as more than a mechanical assessment tool (Kojima et al., 2021; Liao et al., 2024; Munnukka et al., 2022). Telepresence, by contrast, concerns candidates' sense of involvement in the mediated interview environment, including whether the process feels coherent, structured, and engaging enough to hold their attention (Lombard and Ditton, 1997; Steuer, 1992). An AI interviewer may therefore feel socially responsive without making candidates feel strongly immersed in the interview environment, just as a structured interview interface may feel involving without necessarily conveying interpersonal warmth. Distinguishing these two forms of presence allows the present study to examine whether organizational attractiveness is shaped primarily by perceived social connectedness, by involvement in the mediated interview environment, or by both.

In AI-mediated interviews, candidates encounter a consequential evaluator whose technical logic is largely hidden. They therefore rely on visible design cues to infer what kind of interaction they are entering and what the hiring organization communicates through the system. This premise is consistent with prior research suggesting that people respond to technological agents and interfaces according to the social and informational cues they perceive (Epley et al., 2007; Qiu and Benbasat, 2009). The present study focuses on three perceived design features: human-likeness cues, decision transparency, and feedback informativeness. These features are conceptually distinct because they speak to different aspects of the interview encounter. Human-likeness cues indicate the social character of the AI interviewer, decision transparency concerns the intelligibility of the evaluation process, and feedback informativeness reflects the communicative value of information offered about evaluation criteria, performance, or outcomes. The selection of these features follows from the candidate's position in an AI-mediated evaluative encounter. A candidate must make sense of who or what is interacting with them, how their responses are being judged, and whether the system provides meaningful information in return. Human-likeness cues, decision transparency, and feedback informativeness map onto these three interpretive tasks and are therefore treated as social, explanatory, and communicative cues. Other attributes, such as response speed or emotional support, may also affect candidate reactions, but they are more directly tied to system usability or affective assistance. They fall outside the present model, which centers on design features that carry organizational meaning by shaping how candidates experience an AI interview as both an assessment procedure and a mediated interaction.

Human-likeness cues refer to the extent to which an AI interviewer is perceived as displaying human-like characteristics. Research on anthropomorphism suggests that when artificial agents are perceived as more human-like, individuals are more likely to interpret them in social rather than purely technical terms (Epley et al., 2007; Qiu and Benbasat, 2009). Such perceptions may be shaped by interface design, visual representation, and interactional style (Gong, 2008; van Pinxteren et al., 2019). In AI-mediated interviews, these cues are expected to be especially relevant to social presence because a more human-like interviewer may make the interaction feel more attentive, responsive, and socially meaningful, rather than merely mechanical (Munnukka et al., 2022; Yoganathan et al., 2021). Human-likeness cues may also relate to telepresence, although this association is likely to be weaker than their relationship with social presence because anthropomorphic cues primarily signal interpersonal qualities rather than environmental immersion (Kim et al., 2023).

**H1a**: AI interviewer human-likeness cues are positively associated with candidates' perceived social presence.
**H1b**: AI interviewer human-likeness cues are positively associated with candidates' perceived telepresence.

Decision transparency refers to the extent to which candidates can understand how an AI interview system evaluates responses and generates outcomes. In AI-based contexts, transparency reduces the opacity that often characterizes algorithmic decision-making and helps users make better sense of how the system operates (Ananny and Crawford, 2018; Grimmelikhuijsen, 2023; Suen and Hung, 2023). Prior research has also shown that opacity can intensify concerns about accountability, trustworthiness, and fairness, especially in high-stakes settings such as hiring (Bitzer et al., 2023; Raghavan et al., 2020). In AI-mediated interviews, transparency may strengthen social presence not because explanations make the system human-like, but because they make the evaluative encounter more intelligible and accountable. When candidates understand how their responses are assessed, the AI interviewer is less likely to be experienced as an opaque evaluator operating beyond the candidate's reach. Instead, the system may be perceived as a more responsive interactional counterpart that provides reasons for its judgments. Because responsiveness and acknowledgment are central to social presence in mediated interactions, decision transparency can support a stronger sense of social connection within the AI-mediated interview. Transparency may also support telepresence by reducing uncertainty and helping candidates remain cognitively engaged with the interview process rather than distracted by doubts about how the system works.

**H2a**: AI interview decision transparency positively affects candidates' perceived social presence.
**H2b**: AI interview decision transparency positively affects candidates' perceived telepresence.

Feedback informativeness refers to the extent to which an AI interview provides useful and meaningful information about evaluation criteria, performance, or outcomes. In recruitment contexts, informative feedback has long been associated with more favorable candidate reactions because it signals respect, consideration, and procedural care (Gilliland, 1993; Tyler and Bies, 2015). Candidates also value feedback that helps them interpret selection outcomes and understand how they were assessed, particularly when technology is involved in the evaluation process (Mirowska and Mesnet, 2022). In AI-mediated interviews, informative feedback may be especially relevant to social presence because meaningful feedback can make the system appear more responsive and less indifferent to the individual. Feedback informativeness may contribute more directly to social presence than to telepresence. Although useful feedback may help candidates interpret the interview as a more coherent interaction, its connection to immersion is likely to be less direct (Anseel and Lievens, 2009; Kizilcec, 2016).

**H3a**: AI interview feedback informativeness positively affects candidates' perceived social presence.
**H3b**: AI interview feedback informativeness positively affects candidates' perceived telepresence.

Candidates' experiences during selection processes often shape how they evaluate organizations as potential employers (Turban and Keon, 1993). In AI-mediated interviews, social presence and telepresence may matter both as responses to interview design and as antecedents of organizational attractiveness. When the interview feels socially meaningful or involving, candidates may form more favorable impressions of the organization using the system.

**H4a:** Social presence positively affects organizational attractiveness.
**H4b:** Telepresence positively affects organizational attractiveness.

Perceived presence may serve as a central mechanism linking AI interview design features to organizational attractiveness. In AI-mediated interviews, candidates do not evaluate system features solely in procedural terms. They also form impressions from the quality of the mediated interaction itself. Social presence and telepresence are therefore treated as experiential states through which interview design features may shape evaluations of the hiring organization.

AI interviewer human-likeness cues may influence organizational attractiveness through perceived presence. When the AI interviewer is perceived as more human-like, the interaction may feel less mechanical and more socially engaging, which can enhance organizational attractiveness (Turban and Keon, 1993).

**H5a**: Social presence mediates the relationship between AI interviewer human-likeness cues and organizational attractiveness.
**H5b**: Telepresence mediates the relationship between AI interviewer human-likeness cues and organizational attractiveness.

AI interview decision transparency may influence organizational attractiveness through perceived presence. When candidates understand how the system evaluates responses and produces outcomes, the interview can become more intelligible as both an assessment procedure and a mediated interaction. This intelligibility may foster social presence by making the system appear more responsive and accountable, and it may foster telepresence by helping candidates remain oriented within the interview environment. These experiential responses may then shape broader evaluations of the hiring organization.

**H6a**: Social presence mediates the relationship between AI interview decision transparency and organizational attractiveness.
**H6b**: Telepresence mediates the relationship between AI interview decision transparency and organizational attractiveness.

AI interview feedback informativeness may influence organizational attractiveness through perceived presence. When feedback is perceived as useful and meaningful, candidates may view the standardized interview setting as more responsive and considerate, rather than impersonal or indifferent. This is likely to strengthen social presence. Feedback informativeness may also shape the overall interview experience, although its connection to telepresence may be less direct.

**H7a**: Social presence mediates the relationship between AI interview feedback informativeness and organizational attractiveness.
**H7b**: Telepresence mediates the relationship between AI interview feedback informativeness and organizational attractiveness.

As AI-mediated recruitment becomes more common, candidates increasingly encounter interview settings in which they must interpret not only the content of the interview, but also the meaning of the AI-enabled interaction itself. AI literacy therefore represents an important individual difference in this context. Broadly, AI literacy refers to individuals' capacity to understand, critically evaluate, and effectively use AI systems in a given context (Pinski and Benlian, 2024). In AI-mediated assessment settings, such literacy may shape how individuals interpret and respond to technology-enabled experiences (Yilmaz Soylu et al., 2025).

The present study positions AI literacy as a later-stage interpretive boundary condition rather than as a moderator of the initial effects of design features on perceived presence. This placement follows from the distinction between experiencing an AI-mediated interview and interpreting what that experience implies about the organization. The three design features examined in this study are visible candidate-facing cues that are expected to shape social presence and telepresence directly. By contrast, organizational attractiveness requires candidates to translate these experiential states into broader judgments about the hiring organization. Candidates with higher AI literacy may be better able to understand what an AI-mediated interview experience signals about the organization's technological competence, assessment approach, and candidate orientation. They may therefore be more likely to convert a meaningful or involving AI-mediated interview experience into favorable organizational evaluations. Candidates with lower AI literacy may still experience social presence or telepresence, but they may be less able to interpret these experiences as informative organizational signals. This reasoning is consistent with research suggesting that AI-related literacies shape how individuals interpret and respond to technology-enabled experiences (Tulcanaza-Prieto et al., 2025; Yuan et al., 2025).

**H8a**: AI literacy positively moderates the relationship between social presence and organizational attractiveness.
**H8b**: AI literacy positively moderates the relationship between telepresence and organizational attractiveness.

This study is grounded in the stimulus–organism–response (S–O–R) framework, which explains how environmental cues shape individuals' internal states and subsequent evaluative reactions or behavioral intentions (Mehrabian and Russell, 1974). In this framework, stimuli refer to external environmental cues, organism states capture individuals' internal cognitive and affective responses, and responses reflect the resulting evaluative reactions or behavioral intentions. The S–O–R framework has been widely applied in consumer behavior, information systems, and human–computer interaction research and more recently extended to AI-mediated recruitment contexts. Prior S–O–R-based research shows that AI interviewer anthropomorphism and decision transparency can influence candidates' fairness perceptions, which subsequently shape satisfaction with AI interviews and organizational recommendation intentions (Ochmann et al., 2024). This prior work also suggests that justice- and trust-related evaluations can be incorporated within an S–O–R logic, but the present study extends the framework by focusing on presence-based experiential states rather than fairness or trust perceptions as the central organism mechanism. These findings demonstrate the usefulness of the S–O–R framework in explaining how AI interview design features shape candidate outcomes.

Building on this perspective, the present study develops a presence-based S–O–R model of candidate evaluation in AI-mediated interviews. Specifically, AI interview design features—AI interviewer human-likeness cues, decision transparency, and feedback informativeness—are conceptualized as key stimuli. Perceived presence is positioned as the central organism component and is represented by two experiential states: social presence and telepresence. As discussed above, social presence and telepresence represent two distinct organism states in the proposed model: the former captures the perceived social connectedness of the AI-mediated interaction, whereas the latter captures candidates' involvement in the mediated interview environment. Organizational attractiveness is specified as the response variable, representing candidates' overall evaluation of an organization as a desirable place to work.

**Figure 1:**
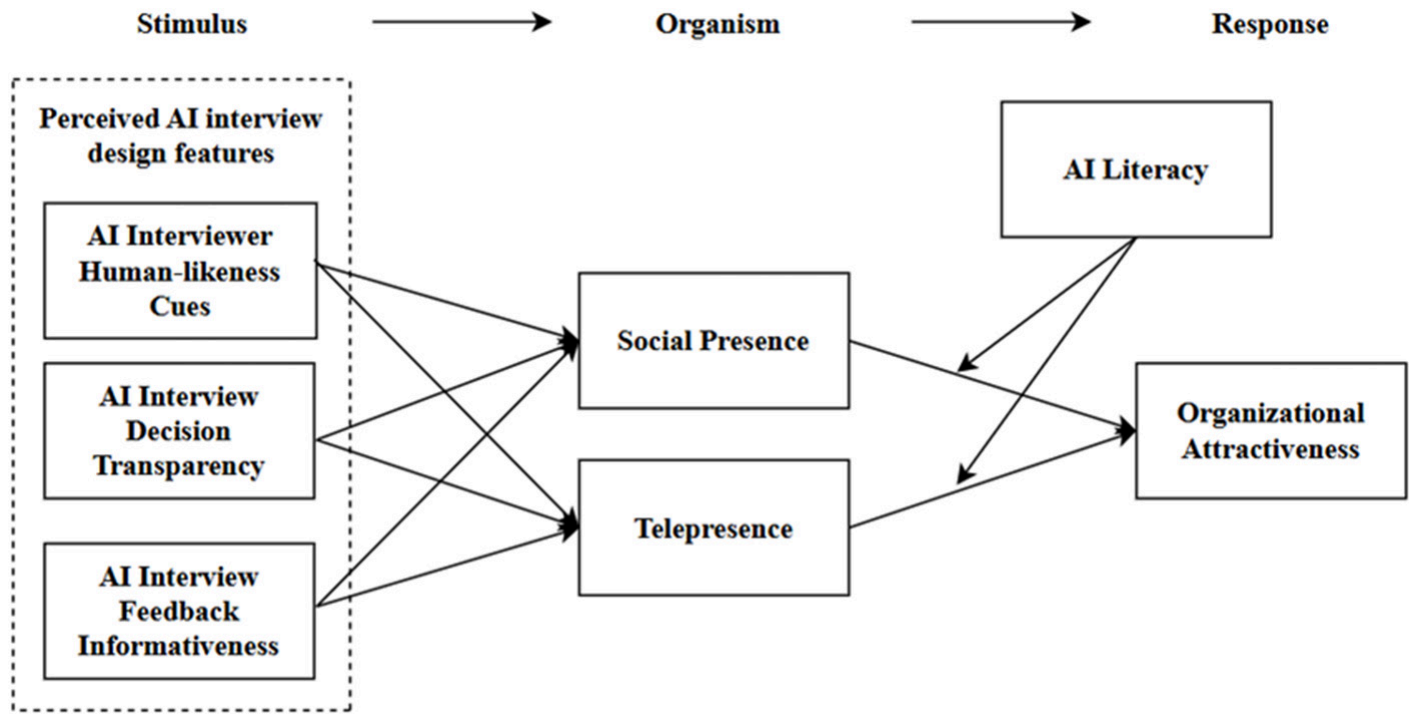
Presence-based S–O–R model of perceived AI interview design and organizational attractiveness.

The model further proposes that AI literacy serves as an interpretive boundary condition at the response stage of the S–O–R process. In this model, AI interview design features first shape candidates' anticipated experiential states, represented by social presence and telepresence. AI literacy is then expected to condition how these experiential states are translated into organizational attractiveness, reflecting individual differences in candidates' ability to interpret AI-mediated interview experiences as organizational signals. The full theoretical model and hypothesized relationships are presented in Figure 1.

## 2 Methodology

### 2.1 Research design and data collection

This study employed a scenario-based questionnaire survey to examine how candidates evaluate key design features of a standardized AI-mediated interview and how those perceptions relate to perceived presence and organizational attractiveness. Guided by the proposed presence-based S–O–R framework, a structured questionnaire was developed to measure AI interviewer human-likeness cues, decision transparency, feedback informativeness, social presence, telepresence, AI literacy, and organizational attractiveness. All measurement items were assessed on a seven-point Likert scale ranging from 1 (“strongly disagree”) to 7 (“strongly agree”). Before completing the questionnaire, respondents were presented with a standardized scenario describing an AI-mediated first-round interview in which an AI interviewer asked structured questions, recorded candidates' video responses, evaluated performance, and generated automated feedback. Participants were instructed to read the scenario carefully and answer all questionnaire items based on that interview context. To make the scenario more concrete and easier to follow, Appendix B provides screenshots of the AI interview interface, including the AI interviewer interface, a feedback example, and an illustration of the evaluation criteria. The scenario-based design was used to provide respondents with a common frame of reference. This choice was important because actual AI-mediated interview systems differ substantially in interface design, feedback functions, evaluation logic, and levels of transparency. Asking respondents to evaluate their own prior experiences could therefore have introduced uncontrolled variation across different systems and recruitment contexts. By presenting a standardized scenario, the study isolated candidates' perceptions of the focal design features and allowed the proposed S–O–R process to be tested under a consistent interview context. At the same time, the scenario was designed to approximate a realistic first-round AI interview by describing structured questions, video response recording, AI-based evaluation, and automated feedback, supported by screenshots of the interview interface, feedback example, and evaluation criteria. The findings should therefore be interpreted as candidates' anticipated evaluations of a standardized AI-mediated interview rather than as real-time reactions observed during a live selection encounter. The survey was administered online through a professional survey platform. The target respondents were candidates with prior job-search experience and familiarity with online recruitment platforms. Although some respondents had previous exposure to technology-mediated or AI-supported recruitment procedures, all participants were instructed to evaluate the same standardized AI interview scenario presented in the survey rather than any specific past interview experience. After excluding incomplete responses and removing careless or patterned answers, 755 valid questionnaires were retained for analysis. The final sample covered a range of demographic backgrounds and provided sufficient variation for the subsequent analyses. The demographic characteristics of the respondents are presented in Table 1. The findings should therefore be interpreted as candidates' anticipated evaluations of a standardized AI-mediated interview context rather than real-time reactions observed during a live interview.

**Table 1:** Demographic characteristics of the sample (*N* = 755).

Variable	Category	Frequency	Percentage (%)
Gender	Male	374	49.54
	Female	381	50.46
Age	20–29 years	363	48.08
	30–39 years	193	25.56
	40–49 years	98	12.98
	50–59 years	71	9.40
	60 years and above	30	3.97
Highest education level	High school or below	190	25.17
	Associate degree	191	25.30
	Bachelor's degree	180	23.84
	Master's degree or above	194	25.70
Academic background	Science and engineering	122	16.16
	Economics and management	131	17.35
	Humanities and languages	137	18.15
	Law and social sciences	114	15.10
	Education and arts	132	17.48
	Medicine and life sciences	119	15.76

### 2.2 Scale development and pretest validation

To assess the reliability and validity of the measures, item analysis and exploratory factor analysis were conducted before hypothesis testing. Using the pilot sample (*N* = 157), all items were assessed for reliability and construct validity. Most measurement items were adapted from established scales and revised where necessary to fit the AI-mediated interview context of the present study. The initial questionnaire included 30 items representing seven constructs. AI interviewer human-likeness cues were adapted from Bartneck et al. (2009), decision transparency from Liu et al. (2015), feedback informativeness from Park et al. (2007), social presence from Verhagen et al. (2014), telepresence from Lombard and Ditton (1997), AI literacy from Pinski and Benlian (2024) and Yilmaz Soylu et al. (2025), and organizational attractiveness from Highhouse et al. (2003). Full item wording and sources are reported in Appendix Table A1. The AI literacy scale was used as a self-report measure of candidates' perceived capacity to understand, critically evaluate, and interpret AI-based interview systems. It was not intended to assess objective AI knowledge through performance-based questions or technical tests. This measurement decision is important because recent research suggests that AI literacy may be related to AI receptivity in ways that differ from intuitive assumptions about knowledge and acceptance of AI (Tully et al., 2025).

A total of 157 valid responses were retained for pretest analysis. Item screening began with critical ratio analysis and item–total correlation analysis. For the critical ratio test, respondents were divided into upper and lower groups based on the top and bottom 27% of the total score distribution. Independent samples t-tests showed that all items significantly distinguished between the two groups (p < 0.01), with t-values above the recommended threshold. Item–total correlation analysis further indicated that most items were adequately correlated with their respective total scale scores. One item, HLC4, showed a relatively low item–total correlation (*r* = 0.357), below the recommended cutoff of 0.40, and was therefore removed. All remaining items exceeded the 0.40 threshold, indicating acceptable item reliability.

Reliability analysis showed good internal consistency across all constructs. Cronbach's alpha coefficients were above 0.80 for every scale, and all corrected item–total correlations exceeded 0.40. Removing any additional item did not produce a meaningful increase in Cronbach's alpha, suggesting that the retained items formed stable and reliable scales. Exploratory factor analysis was then conducted using principal component extraction with Varimax rotation. The data showed good sampling adequacy (KMO = 0.868) and a significant Bartlett's test of sphericity (χ^2^ = 2,518.56, *p* < 0.001), supporting the suitability of the data for factor analysis. The initial EFA extracted seven factors with eigenvalues greater than one, accounting for 70.38% of the total variance. Examination of the rotated factor matrix showed that SP5 loaded above 0.40 on two factors, indicating a cross-loading problem. To obtain a cleaner factor structure, SP5 was removed and the analysis was rerun with the remaining 28 items. The second EFA again supported a clear seven-factor solution (KMO = 0.867; Bartlett's χ^2^ = 2,397.21, *p* < 0.001), with the factors explaining 70.91% of the total variance. All retained items loaded strongly on their intended factors, with standardized loadings above 0.50 and no problematic cross-loadings.

The pretest results support the reliability and construct validity of the measures. After item purification, the final scales showed satisfactory internal consistency and a clear seven-factor structure corresponding to the proposed constructs: AI interviewer human-likeness cues, decision transparency, feedback informativeness, social presence, telepresence, AI literacy, and organizational attractiveness. These refined scales were used in the main study. In addition to assessing reliability and factor structure, the pilot test helped confirm the clarity and contextual appropriateness of the adapted items.

### 2.3 Measurement model and construct validity

Using the main survey data (*N* = 755), confirmatory factor analysis (CFA) was conducted in Mplus 8.3 to assess the reliability and validity of the measurement model. The model comprised seven latent constructs: AI interviewer human-likeness cues (HLC), decision transparency (DT), feedback informativeness (FI), social presence (SP), telepresence (TP), AI literacy (AL), and organizational attractiveness (OA).

The CFA results indicate that the measurement model fits the data well. The chi-square statistic was χ^2^ = 442.60 with 329 degrees of freedom, yielding a χ^2^/df ratio of 1.35, which is well below the commonly used upper threshold of 5.00. The remaining fit indices also support good model fit (RMSEA = 0.021, CFI = 0.990, TLI = 0.988, SRMR = 0.030). These results indicate that the proposed seven-factor measurement model fits the data well.

Convergent validity was assessed using standardized factor loadings, composite reliability (CR), and average variance extracted (AVE). As shown in Table 2, all standardized factor loadings were statistically significant (*p* < 0.001) and generally above 0.70, indicating that the observed indicators adequately reflected their intended latent constructs. Composite reliability values ranged from 0.829 to 0.890, exceeding the recommended threshold of 0.70 and supporting satisfactory internal consistency. AVE values ranged from 0.574 to 0.660, all above the recommended cutoff of 0.50. These results provide evidence of adequate convergent validity, indicating that the constructs explained a substantial proportion of variance in their corresponding indicators.

**Table 2:** Confirmatory factor analysis results: standardized loadings, composite reliability, and average variance extracted.

Construct	Item	Standardized loading	CR	AVE
Human-likeness cues (HLC)	HLC1	0.838	0.829	0.619
	HLC2	0.711		
	HLC3	0.805		
Decision transparency (DT)	DT1	0.793	0.844	0.643
	DT2	0.766		
	DT3	0.845		
Feedback informativeness (FI)	FI1	0.810	0.853	0.660
	FI2	0.849		
	FI3	0.777		
Social presence (SP)	SP1	0.777	0.879	0.644
	SP2	0.828		
	SP3	0.790		
	SP4	0.814		
Telepresence (TP)	TP1	0.805	0.890	0.618
	TP2	0.789		
	TP3	0.778		
	TP4	0.780		
	TP5	0.779		
AI literacy (AL)	AL1	0.803	0.882	0.599
	AL2	0.802		
	AL3	0.743		
	AL4	0.793		
	AL5	0.726		
Organizational attractiveness (OA)	OA1	0.790	0.870	0.574
	OA2	0.731		
	OA3	0.735		
	OA4	0.775		
	OA5	0.754		

Discriminant validity was assessed using the Fornell–Larcker criterion, which compares the square root of each construct's AVE with the correlations between constructs. As shown in Table 3, the square root of AVE for each construct exceeds the corresponding inter-construct correlations. This comparison indicates that each construct shares more variance with its own indicators than with other constructs, thereby supporting the discriminant validity of the measurement model.

**Table 3:** Discriminant validity assessment (Fornell–Larcker criterion).

Construct	HLC	DT	FI	SP	TP	AL	OA
HLC	**0.787**						
DT	0.427^**^	**0.802**					
FI	0.457^**^	0.436^**^	**0.812**				
SP	0.363^**^	0.393^**^	0.438^**^	**0.802**			
TP	0.275^**^	0.431^**^	0.252^**^	0.333^**^	**0.786**		
AL	0.291^**^	0.303^**^	0.232^**^	0.295^**^	0.429^**^	**0.774**	
OA	0.415^**^	0.389^**^	0.360^**^	0.344^**^	0.302^**^	0.333^**^	**0.758**

Diagonal elements (bold) represent the square root of AVE. Off-diagonal elements represent Pearson correlations. ^**^p < 0.01. Bold values represent the square root of AVE for each construct.

To further strengthen the assessment of discriminant validity, the heterotrait–monotrait ratio of correlations (HTMT) was examined as an additional check. All HTMT values ranged from 0.267 to 0.545, below the commonly used threshold of 0.85. The HTMT value between social presence and telepresence was 0.377, providing additional evidence that the two forms of presence were empirically distinguishable. The HTMT values among the three perceived design features ranged from 0.509 to 0.545, indicating that human-likeness cues, decision transparency, and feedback informativeness were also empirically distinguishable despite their significant correlations. These results suggest that candidates may evaluate related aspects of the same AI-mediated interview experience without treating them as redundant indicators of a single underlying construct.

### 2.4 Common method bias

Because all focal variables were collected from the same respondents in a single survey, common method bias was addressed through both procedural and statistical checks. Procedurally, the survey was administered anonymously, respondents were informed that there were no right or wrong answers, and the standardized scenario provided a common reference point before the measurement items were completed. These steps were intended to reduce evaluation apprehension and lessen the likelihood that responses were driven by a single response tendency.

Statistically, three checks were conducted. First, Harman's single-factor test showed that the first unrotated factor accounted for 31.81% of the total variance, below the conventional 40% threshold. Second, the confirmatory factor analysis showed that the hypothesized multi-factor measurement model fit the data well, which is inconsistent with the assumption that the observed relationships were dominated by a single common source. Third, a full collinearity assessment was conducted as an additional diagnostic check. Each focal construct was regressed on the remaining constructs, and the resulting variance inflation factors ranged from 1.333 to 1.576, well below the commonly used threshold of 3.3. These results do not eliminate the possibility of common method bias, but they suggest that it is unlikely to account for the observed pattern of relationships. This interpretation is also consistent with the moderate correlations among the three perceived design features and the HTMT results reported above, which suggest that the three predictors were empirically distinguishable rather than reducible to a single response pattern.

### 2.5 Ethics statement

Participation in this study was voluntary and anonymous. Electronic informed consent was obtained from all participants before participation. No personally identifiable information was collected. In accordance with local regulations and institutional requirements, formal ethical approval was not required for this anonymous minimal-risk survey study.

## 3 Results

### 3.1 Descriptive statistics and correlations

Descriptive statistics and Pearson correlations for the study variables are reported in Table 4. Across the 755 valid responses, mean scores ranged from 4.553 to 5.090 on a seven-point scale. AI literacy and telepresence showed slightly higher mean levels than the other constructs, although the differences were modest (*M* = 5.090 and *M* = 5.032, respectively). Organizational attractiveness was also comparatively high (*M* = 4.979, SD = 1.225). The remaining variables—human-likeness cues (*M* = 4.735, SD = 1.325), feedback informativeness (*M* = 4.688, SD = 1.442), social presence (*M* = 4.799, SD = 1.451), and decision transparency (*M* = 4.553, SD = 1.381)—were likewise close to the midpoint of the scale.

**Table 4:** Descriptive statistics and correlations (*N* = 755).

Variable	Mean	SD	HLC	DT	FI	SP	TP	AL	OA
HLC	4.735	1.325	1						
DT	4.553	1.381	0.427^**^	1					
FI	4.688	1.442	0.457^**^	0.436^**^	1				
SP	4.799	1.451	0.363^**^	0.393^**^	0.438^**^	1			
TP	5.032	1.316	0.275^**^	0.431^**^	0.252^**^	0.333^**^	1		
AL	5.090	1.341	0.291^**^	0.303^**^	0.232^**^	0.295^**^	0.429^**^	1	
OA	4.979	1.225	0.415^**^	0.389^**^	0.360^**^	0.344^**^	0.302^**^	0.333^**^	1

^**^p < 0.01.

The correlation matrix shows that all focal variables were positively related in the expected directions. Human-likeness cues were positively correlated with decision transparency (*r* = 0.427, *p* < 0.01), feedback informativeness (*r* = 0.457, *p* < 0.01), social presence (*r* = 0.363, *p* < 0.01), telepresence (*r* = 0.275, *p* < 0.01), AI literacy (*r* = 0.291, *p* < 0.01), and organizational attractiveness (*r* = 0.415, *p* < 0.01). Decision transparency was also positively associated with feedback informativeness (*r* = 0.436, *p* < 0.01), social presence (*r* = 0.393, *p* < 0.01), telepresence (*r* = 0.431, *p* < 0.01), AI literacy (*r* = 0.303, *p* < 0.01), and organizational attractiveness (*r* = 0.389, *p* < 0.01).

Social presence and telepresence were both positively correlated with organizational attractiveness (*r* = 0.344 and *r* = 0.302, respectively, *p* < 0.01). AI literacy was likewise positively associated with organizational attractiveness (*r* = 0.333, *p* < 0.01). These zero-order correlations were consistent with the proposed model and provided initial support for the structural analyses.

Although the three perceived design features were significantly interrelated, the correlations were moderate rather than high. This pattern suggests that candidates evaluated human-likeness cues, decision transparency, and feedback informativeness as related but nonredundant aspects of the same AI-mediated interview setting. This interpretation is consistent with the additional HTMT results reported in Section 2.3, where the HTMT values among the three design features ranged from 0.509 to 0.545, well below the 0.85 threshold.

### 3.2 Structural model testing

After establishing the adequacy of the measurement model, the structural model was estimated to test the hypothesized relationships among the study constructs. The model showed good fit to the data (χ^2^ = 620.04, df = 335, χ^2^/df = 1.85, CFI = 0.974, TLI = 0.971, RMSEA = 0.034, SRMR = 0.065), indicating that the framework provided an adequate representation of the relationships among the variables. The standardized path coefficients are reported in Table 5.

**Table 5:** Structural equation modeling results: direct effects.

Path	Standardized coefficient (β)	*R^2^*
Predicting social presence (SP)		0.343
HLC → SP	0.156^**^	
DT → SP	0.249^***^	
FI → SP	0.305^***^	
Predicting telepresence (TP)		0.281
HLC → TP	0.095	
DT → TP	0.474^***^	
FI → TP	0.010	
Predicting organizational attractiveness (OA)		0.234
SP → OA	0.290^***^	
TP → OA	0.153^**^	
AI Literacy (AL) → OA	0.234^***^	

^*^p < 0.05, ^**^p < 0.01, ^***^p < 0.001.

Human-likeness cues positively predicted social presence (β = 0.156, *p* < 0.01), supporting **H1a**. Decision transparency also had a significant positive effect on social presence (β = 0.249, *p* < 0.001), supporting **H2a**, while feedback informativeness showed the strongest positive effect on social presence (β = 0.305, *p* < 0.001), supporting **H3a**. These three predictors explained 34.3% of the variance in social presence (*R*^2^ = 0.343). For telepresence, decision transparency had a significant positive effect (β = 0.474, *p* < 0.001), supporting **H2b**. By contrast, the effects of human-likeness cues (β = 0.095, *p* > 0.05) and feedback informativeness (β = 0.010, *p* > 0.05) were not significant; therefore, **H1b** and **H3b** were not supported. These predictors explained 28.1% of the variance in telepresence (*R*^2^ = 0.281). With respect to organizational attractiveness, social presence had a significant positive effect (β = 0.290, *p* < 0.001), supporting **H4a**, and telepresence also had a significant positive effect (β = 0.153, *p* < 0.01), supporting **H4b**. AI literacy was also positively associated with organizational attractiveness (β = 0.234, *p* < 0.001).

### 3.3 Mediation analysis

To examine whether perceived presence mediated the relationships between AI interview design features and organizational attractiveness, indirect effects were estimated using bias-corrected bootstrapping with 5,000 resamples in Mplus 8.3, Muthén & Muthén, Los Angeles, CA, United States. As shown in Table 6, social presence emerged as the more consistent mediator. The indirect effect of human-likeness cues on organizational attractiveness through social presence was significant (β = 0.044, *p* = 0.007), supporting **H5a**. Decision transparency also showed a significant indirect effect through social presence (β = 0.071, *p* < 0.001), supporting **H6a**. Feedback informativeness likewise had a significant indirect effect through social presence (β = 0.080, *p* < 0.001), supporting **H7a**. These findings indicate that social presence serves as a key pathway linking perceived interview design features to organizational attractiveness.

**Table 6:** Bootstrapped indirect effects for the mediation model.

Indirect path	Indirect effect (β)	*SE*	*z*	*p*
HLC → SP → OA	0.044	0.016	2.695	0.007
DT → SP → OA	0.071	0.019	3.684	< 0.001
FI → SP → OA	0.080	0.021	3.800	< 0.001
HLC → TP → OA	0.014	0.011	1.263	0.206
DT → TP → OA	0.071	0.026	2.746	0.006
FI → TP → OA	0.001	0.009	0.150	0.880

Indirect effects were estimated using bias-corrected bootstrap procedures (95% confidence intervals).

The evidence for telepresence was more limited. Decision transparency showed a significant indirect effect on organizational attractiveness through telepresence (β = 0.071, *p* = 0.006), supporting **H6b**. By contrast, the indirect effects of human-likeness cues (β = 0.014, *p* = 0.206) and feedback informativeness (β = 0.001, *p* = 0.880) through telepresence were not significant; thus, **H5b** and **H7b** were not supported. These results suggest that telepresence did not operate as a general mediating mechanism across all three design features, but instead functioned as a more selective pathway that became relevant primarily when the interview system was experienced as understandable and cognitively engaging.

**Figure 2:**
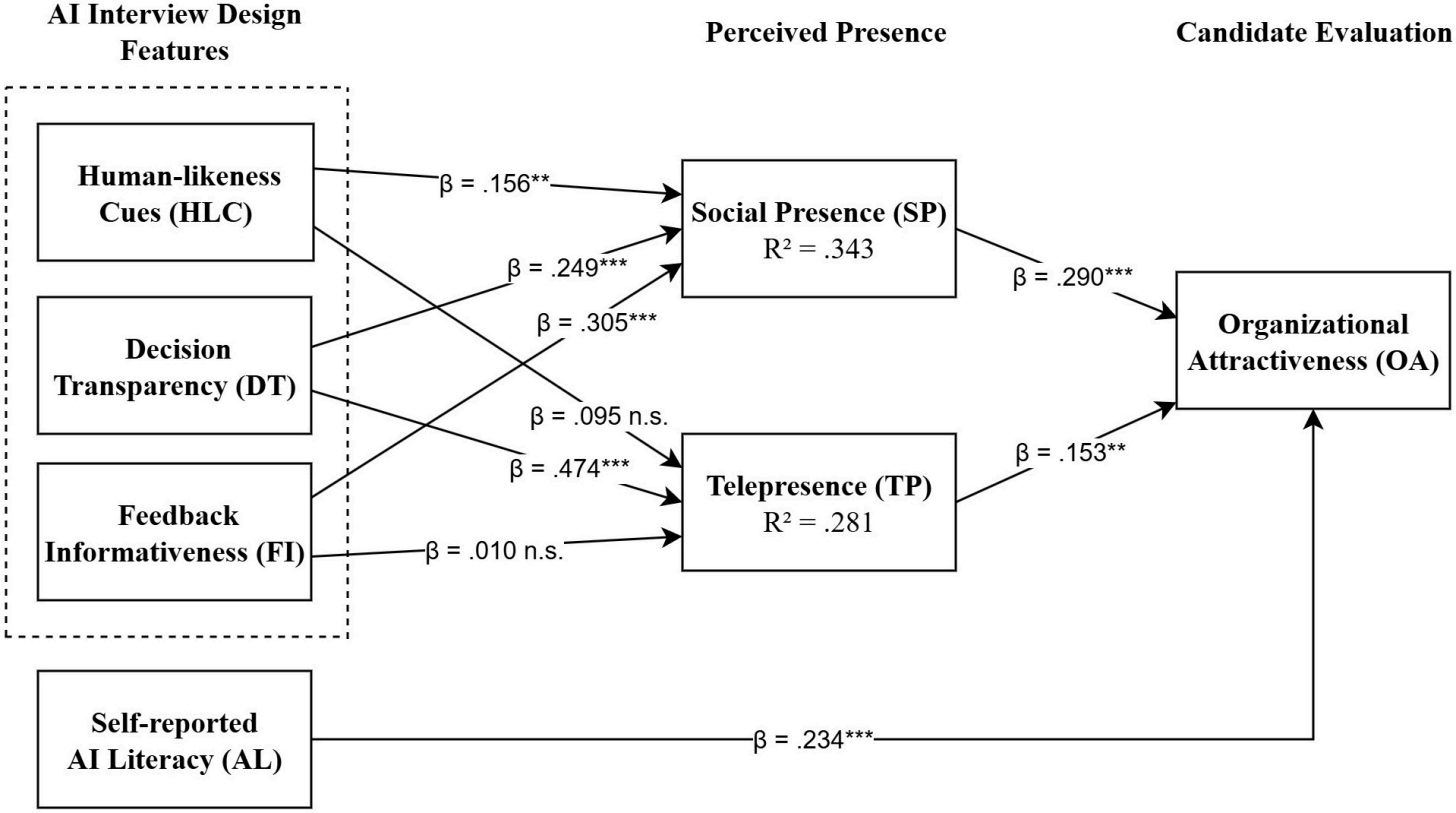
Structural model of the mediation relationships.

Figure 2, presents the structural mediation model, illustrating how AI interview design features are associated with perceived presence and, in turn, organizational attractiveness, with self-reported AI literacy included as an additional predictor of organizational attractiveness.

### 3.4 Moderation analysis

To test whether AI literacy conditions the relationships between perceived presence and organizational attractiveness, a latent interaction model was estimated using the latent moderated structural equations (LMS) approach in Mplus 8.3. As shown in Table 7, the interaction between social presence and AI literacy was not significant (β = −0.061, *p* = 0.293); therefore, **H8a** was not supported. In contrast, the interaction between telepresence and AI literacy was positive and significant (β = 0.183, *p* = 0.001), supporting **H8b**. This pattern suggests that telepresence carries greater evaluative significance for candidates with higher AI literacy. Immersive involvement in the AI-mediated interview appears more likely to translate into favorable organizational evaluations when candidates are better able to interpret AI-based interaction.

**Table 7:** Latent interaction effects on organizational attractiveness.

Interaction path	Standardized coefficient (β)	*SE*	*z*	*p*
SP × AI Literacy → OA	−0.061	0.058	−1.051	0.293
TP × AI Literacy → OA	0.183	0.057	3.219	0.001

**Figure 3:**
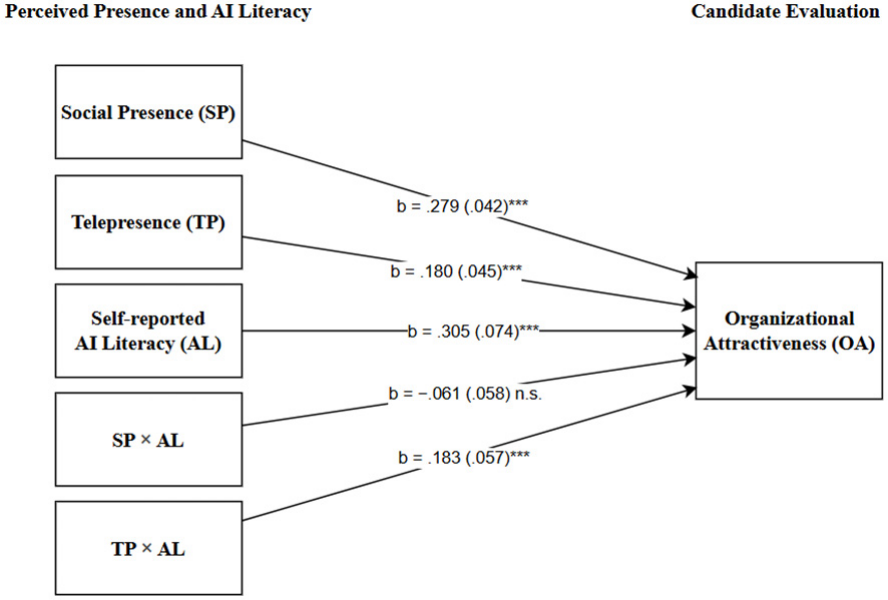
Moderating effects of self-reported AI literacy on organizational attractiveness.

Figure 3, presents the moderation model used to examine whether self-reported AI literacy conditions the effects of social presence and telepresence on organizational attractiveness.

**Figure 4:**
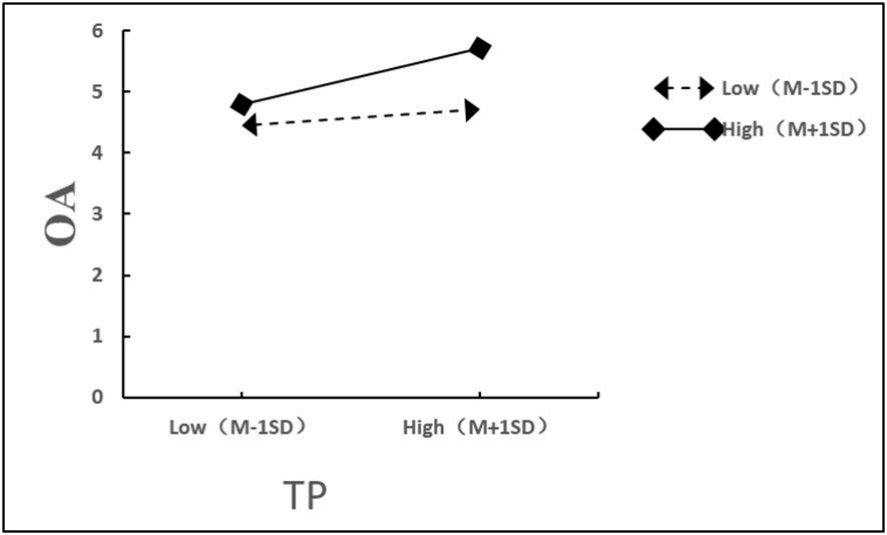
Simple slope of telepresence × AI literacy on organizational attractiveness.

To further interpret the significant interaction effect, a simple slope analysis was conducted. As shown in Figure 4, the positive relationship between telepresence and organizational attractiveness was stronger at higher levels of AI literacy and weaker at lower levels. This result further supports the moderating role of AI literacy.

The overall hypothesis testing results are summarized in Table 8.

**Table 8:** Summary of hypothesis testing results.

Hypothesis	Statement	Result
H1a	AI interviewer human-likeness cues are positively associated with candidates' perceived social presence.	Supported
H1b	AI interviewer human-likeness cues are positively associated with candidates' perceived telepresence.	Not supported
H2a	AI interview decision transparency positively affects candidates' perceived social presence.	Supported
H2b	AI interview decision transparency positively affects candidates' perceived telepresence.	Supported
H3a	AI interview feedback informativeness positively affects candidates' perceived social presence.	Supported
H3b	AI interview feedback informativeness positively affects candidates' perceived telepresence.	Not supported
H4a	Social presence positively affects organizational attractiveness.	Supported
H4b	Telepresence positively affects organizational attractiveness.	Supported
H5a	Social presence mediates the relationship between AI interviewer human-likeness cues and organizational attractiveness.	Supported
H5b	Telepresence mediates the relationship between AI interviewer human-likeness cues and organizational attractiveness.	Not supported
H6a	Social presence mediates the relationship between AI interview decision transparency and organizational attractiveness.	Supported
H6b	Telepresence mediates the relationship between AI interview decision transparency and organizational attractiveness.	Supported
H7a	Social presence mediates the relationship between AI interview feedback informativeness and organizational attractiveness.	Supported
H7b	Telepresence mediates the relationship between AI interview feedback informativeness and organizational attractiveness.	Not supported
H8a	AI literacy positively moderates the relationship between social presence and organizational attractiveness.	Not supported
H8b	AI literacy positively moderates the relationship between telepresence and organizational attractiveness.	Supported

## 4 Discussion

### 4.1 Theoretical implications

This study contributes to research on AI-mediated recruitment by clarifying why candidate reactions to AI interviews cannot be explained only through procedural evaluations. Existing work has given considerable attention to whether AI-based selection is perceived as fair, transparent, trustworthy, or acceptable. Those concerns remain important, but they do not fully capture how candidates experience an AI interview as a mediated organizational encounter. The present findings show that candidates also draw organizational inferences from the felt quality of the interaction itself. When an AI-mediated interview conveys responsiveness, attentiveness, and social meaning, it becomes more than a technical assessment channel; it functions as a recruitment interface through which candidates infer how the organization communicates and treats applicants. This extends applicant reaction research by linking AI-mediated selection to an experience-based account of organizational attractiveness and by showing that social presence is a central mechanism through which such experiences acquire organizational meaning (Kojima et al., 2021; Liao et al., 2024).

The findings also refine the role of presence in AI-mediated interview contexts by explaining why social presence was more dominant than telepresence. Social presence and telepresence are related dimensions of mediated experience, but the present results show that they should not be treated as interchangeable. Social presence provided the more consistent pathway from interview design features to organizational attractiveness, whereas telepresence operated more narrowly and was mainly activated by decision transparency. This asymmetry is theoretically meaningful. AI-mediated interviews are not experienced primarily as immersive digital environments; they are structured, high-stakes social-evaluative encounters in which candidates are being assessed by a technology-mediated system. In such a setting, candidates are likely to attend first to whether the system acknowledges them, responds to them, and makes the interaction feel socially meaningful. Human-likeness cues therefore appear to function mainly as social cues. They may make the AI interviewer seem more attentive or socially responsive, but they do not necessarily make the broader interview environment feel more immersive or cognitively involving. This helps explain why human-likeness cues predicted social presence but did not significantly predict telepresence. A similar logic applies to feedback informativeness, which communicates responsiveness and procedural care but may not, by itself, create a stronger sense of environmental involvement. By contrast, telepresence depends more on whether the interview environment feels coherent and cognitively orienting. Decision transparency may therefore support telepresence by reducing uncertainty about how the AI system operates and helping candidates remain oriented within the interaction (Dalvi-Esfahani et al., 2025). These findings add nuance to presence theory by showing that immersion and social responsiveness carry different explanatory weight in structured, high-stakes, technology-mediated selection settings (Lombard and Ditton, 1997; Pan et al., 2025; Steuer, 1992).

The moderation results further clarify how AI literacy should be understood in this model. Rather than functioning as a general amplifier of all positive AI-related perceptions, AI literacy appears to operate as a selective interpretive condition. It did not strengthen the relationship between social presence and organizational attractiveness, suggesting that socially responsive cues may be interpreted relatively directly, even by candidates with lower perceived AI understanding. By contrast, AI literacy strengthened the effect of telepresence on organizational attractiveness. This result suggests that more technology-mediated forms of involvement require candidates to make sense of the AI-enabled environment before they can translate that experience into favorable organizational evaluations. In theoretical terms, AI literacy is better understood here as a response-stage boundary condition within the S–O–R process: it shapes how candidates interpret the organizational meaning of an AI-mediated experience, rather than simply determining whether they notice design cues or experience presence. This finding adds nuance to AI literacy research by showing that its role depends on the type of mediated experience being interpreted and the evaluative inference candidates are asked to make (Pinski and Benlian, 2024; Tulcanaza-Prieto et al., 2025).

### 4.2 Practical implications

The findings offer several practical implications for organizations that use AI-mediated interviews. These systems should not be treated merely as instruments for efficiency and standardization. From the candidate's perspective, the interview is also part of the organization's recruitment interface and can shape impressions of the employer at a consequential stage of selection. Organizations therefore need to look beyond the technical performance of AI-based evaluation and consider whether the interview process appears clear, responsive, and engaging. In practice, this means designing AI-mediated interviews in ways that minimize impressions of impersonality while maintaining clarity about how the system operates and how candidate responses are assessed. AI-mediated interviews should be managed not simply as assessment technologies, but as recruitment practices that communicate organizational values to prospective candidates.

Among the design features examined in this study, decision transparency appears particularly important. It was the only feature associated with organizational attractiveness through both social presence and telepresence, suggesting that transparency plays a central role in how candidates interpret AI-mediated interviews. Organizations should therefore explain how interview responses are evaluated, what information enters the decision process, and how interview data are stored and used. Transparency is likely to be more effective when it is built into the interview experience itself rather than communicated only through general statements about responsible AI use. Explanations delivered before, during, and after the interview may help candidates understand the system and remain engaged throughout the process. These practical recommendations also carry ethical implications. In AI-mediated interviews, transparency should not be limited to making the system more acceptable to candidates; it should also function as part of a broader governance approach to bias, equity, and accountability. Organizations should clarify what types of data are analyzed, how evaluation criteria are defined, whether human oversight is involved, and how candidates can seek clarification or contest problematic assessments. They should also regularly audit AI interview systems to examine whether evaluation outcomes differ systematically across demographic groups, language styles, disability status, or levels of digital familiarity. Human-likeness cues and feedback functions should not be used to create a misleading sense of personal attention or trustworthiness around algorithmic evaluation. These design features should be accompanied by clear disclosure, accessible explanations, and safeguards to ensure that candidates are not disadvantaged by opaque or unevenly calibrated AI-based assessments. The findings also underscore the importance of communication quality and differences in technological familiarity. Feedback informativeness shaped candidate reactions primarily through social presence, indicating that candidates are especially responsive to cues that make the interview feel communicative and attentive. Even when individualized feedback is not feasible, organizations can still improve candidate perceptions by providing clear guidance on interview procedures, response expectations, and next steps. At the same time, candidates differ in AI literacy, and organizations should not assume that all candidates interpret AI-mediated interviews in the same way. Clear instructions, accessible explanations, and user-friendly guidance may reduce unnecessary uncertainty and help candidates engage with the system with greater confidence.

### 4.3 Limitations and future research directions

First, the study relied on a scenario-based survey rather than actual participation in a live AI-mediated interview. This design allowed all respondents to evaluate the same standardized interview setting and reduced uncontrolled variation arising from differences across existing AI interview systems. However, it also limits external validity. Candidates in a real AI interview may experience stronger pressure, uncertainty, impression-management concerns, time constraints, and emotional involvement than respondents reading a hypothetical scenario. The findings should therefore be interpreted as evidence of anticipated evaluative reactions rather than direct evidence of behavior or experience in live AI interview settings. Relatedly, the outcome variable captures organizational attractiveness as a candidate-side evaluative response rather than observed candidate behavior. Although organizational attractiveness is relevant to recruitment because it reflects whether candidates view an employer as desirable, it does not show whether candidates would actually complete an AI interview, continue an application, recommend the organization, or accept a job offer. Actual behavior may also be shaped by factors outside the interview experience, such as labor-market conditions, alternative job opportunities, economic need, and the perceived value of the position. The present findings should therefore be understood as evidence of how AI-mediated interview perceptions shape organizational evaluations, rather than as direct evidence of downstream behavioral action. Future research could address these limitations through field studies, live AI interview simulations, experimental designs using interactive interview platforms, or longitudinal designs that follow candidates before, during, and after AI-mediated interviews. In a live and interactive setting, the model may need to move from a static cue-based structure to a more dynamic process model. AI interview design features would not be limited to pre-specified perceptions of human-likeness, transparency, and feedback, but could include real-time system responsiveness, conversational contingency, response latency, perceived control, and opportunities for clarification. Social presence and telepresence may also fluctuate during the interview as candidates receive prompts, respond to follow-up questions, encounter delays, or interpret feedback. Future studies could further validate the perception–behavior link by combining survey measures with behavioral data from actual recruitment processes. For example, researchers could examine whether candidates who report higher social presence, telepresence, or organizational attractiveness are more likely to complete an AI interview, continue to later selection stages, accept a follow-up interview invitation, recommend the organization, or accept a job offer. Platform-based studies could also use behavioral logs, such as completion time, dropout points, response latency, requests for clarification, or click-through behavior after feedback. Such designs would allow future research to test whether perceived presence and organizational attractiveness serve as intermediate mechanisms linking AI interview experience to observable recruitment behavior. Second, the model centers on perceived presence and therefore does not capture the full range of psychological processes that may shape candidate reactions to AI-mediated recruitment. The finding that social presence was the more consistent pathway, whereas telepresence played a narrower role, suggests that additional mechanisms may also be involved. Future research could extend the model by incorporating constructs such as perceived fairness, procedural justice, trust in AI systems, perceived algorithmic competence, and interview-related anxiety. Third, although AI literacy was examined as an important source of individual variation, it is unlikely to be the only one that matters. AI literacy was also measured through self-reported perceptions rather than objective knowledge tests. Although this approach fits the study's focus on candidates' perceived interpretive capacity in a scenario-based AI interview setting, it may not fully capture what candidates actually know about AI systems. This distinction matters because recent evidence suggests that AI literacy can relate to AI receptivity in counterintuitive ways, with lower AI literacy sometimes predicting greater receptivity toward AI (Tully et al., 2025). Future research could therefore combine self-reported AI literacy with objective indicators, such as knowledge tests, task-based assessments, or behavioral measures of how candidates understand and use AI-generated information during recruitment. Future research could also examine a broader set of individual differences that may shape how candidates interpret AI-mediated interviews. Digital self-efficacy and prior experience with AI-supported recruitment may influence whether candidates feel capable of navigating the system. AI anxiety, algorithm aversion, and privacy concern may affect whether candidates interpret the same design features as helpful, intrusive, or threatening. Technology readiness, need for human interaction, and perceived employability may also shape how candidates respond to reduced human contact and automated evaluation. These factors could be incorporated as moderators at different stages of the model. For example, some may influence how candidates interpret specific design cues before social presence and telepresence are formed, whereas others may shape how perceived presence is translated into organizational attractiveness or behavioral intentions. Fourth, the data were collected in a single national and cultural context, which may limit the generalizability of the findings. Candidate reactions to AI-mediated interviews may vary across cultures because applicants can differ in their expectations regarding automation, interpersonal treatment, procedural transparency, privacy, and the appropriate role of technology in hiring decisions. In some contexts, a standardized AI interview may be interpreted as efficient and impartial, whereas in others it may be viewed as impersonal, opaque, or insufficiently attentive to individual circumstances. These cultural differences may shape how candidates interpret human-likeness cues, decision transparency, feedback informativeness, and the presence experience generated by AI-mediated interviews. Future research should therefore test the model across different cultural, institutional, and regulatory contexts, including settings with different levels of AI adoption, labor-market competitiveness, privacy regulation, and norms regarding human involvement in personnel selection.

## 5 Conclusions

This study shows that candidates' evaluations of a standardized AI-mediated interview are shaped by both assessment-related features and the quality of the mediated interaction itself. Perceived presence emerged as a key mechanism linking AI interview design to organizational attractiveness, and this process varied partly with candidates' AI literacy. Social presence was the more consistent pathway across the model, whereas telepresence played a narrower and more conditional role. The findings suggest that candidates are especially sensitive to cues of social responsiveness, while immersive involvement becomes more influential when candidates possess higher AI literacy. AI-mediated interviews therefore function as candidate-facing organizational encounters through which candidates form impressions of potential employers. By clarifying how experiential perceptions connect interview design features to organizational attractiveness, this study contributes to a more differentiated understanding of candidate experience in technology-mediated recruitment and offers practical guidance for designing more human-centered and candidate-sensitive AI-mediated interview practices.

## Data Availability

The raw data supporting the conclusions of this article will be made available by the authors, without undue reservation.
