# Supplementary material for: Perceived AI interview design and organizational attractiveness: the roles of social presence, telepresence, and AI literacy
Source: Front Psychol. 2026 Jun 24;17:1836040. doi: 10.3389/fpsyg.2026.1836040 (PMC13342212; doi:10.3389/fpsyg.2026.1836040)
Supplement: Supplementary file 1 [file Supplementary_file_1.docx]

# Appendix A

**Table A1.** Measurement Items and Sources

| Construct | Code | Measurement Item | Adapted From |
| --- | --- | --- | --- |
| AI interviewer human-likeness cues | HLC1 | I feel that this AI interviewer is more human-like rather than machine-like. | Bartneck et al. (2009) |
|  | HLC2 | Compared with traditional automated systems, this AI interviewer feels more human-like. |  |
|  | HLC3 | During the interaction, I perceive this AI interviewer as being similar to a real human interviewer. |  |
| AI Interview Decision Transparency | DT1 | The process by which the AI interviewer arrived at the interview decision was clear. | Liu et al. (2015) |
|  | DT2 | The process by which the AI interviewer arrived at the interview decision was accessible. |  |
|  | DT3 | The process by which the AI interviewer arrived at the interview decision was easy to understand. |  |
| AI Interview Feedback Informativeness | FI1 | The AI interviewer provided feedback on my interview performance from multiple aspects. | Park et al. (2007) |
|  | FI2 | The AI interviewer provided sufficient reasons to explain my interview results. |  |
|  | FI3 | I was able to obtain enough information from the feedback provided by the AI interviewer. |  |
| Social Presence | SP1 | I felt a sense of human contact during the interaction with the AI interviewer. | Verhagen et al. (2014) |
|  | SP2 | I felt as if the AI interviewer was a real person. |  |
|  | SP3 | I felt a sense of interpersonal interaction with the AI interviewer. |  |
|  | SP4 | I felt warmth and friendliness from the AI interviewer. |  |
| Telepresence | TP1 | When interacting with the AI interviewer, I felt as if I were actually present in the interview environment. | Lombard and Ditton (1997) |
|  | TP2 | I had the sensation of “being there” rather than merely observing the interaction through a screen. |  |
|  | TP3 | The interaction experience made me feel as though I had entered a real advisory situation. |  |
|  | TP4 | During the interaction, the mediated environment faded away, and I felt immersed in the experience. |  |
|  | TP5 | I felt psychologically transported into the interaction scenario. |  |
| AI Literacy | AL1 | I understand how AI technologies are used to evaluate candidates in interviews. | Pinski and Benlian (2024); Yilmaz Soylu et al. (2025) |
|  | AL2 | I am able to understand how the AI interviewer analyzes and interprets interview data. |  |
|  | AL3 | I can critically evaluate the interview results generated by an AI system. |  |
|  | AL4 | I feel confident in interpreting decisions made by AI-based interview systems. |  |
|  | AL5 | I am able to judge whether the decisions made by an AI interview system are reasonable. |  |
| Organizational Attractiveness | OA1 | I think this organization would be a good place to work. | Highhouse et al. (2003) |
|  | OA2 | I find this organization attractive as an employer. |  |
|  | OA3 | I am interested in learning more about this organization. |  |
|  | OA4 | I would accept a job offer from this organization. |  |
|  | OA5 | I would consider this organization as one of my future employers. |  |

# Appendix B

Screenshots below illustrate the AI-mediated interview interface presented to respondents in the scenario-based survey. The materials show the AI interviewer avatar, the example of automated feedback, and the explanation of evaluation criteria used by the system.

(Tully et al., 2025)

**
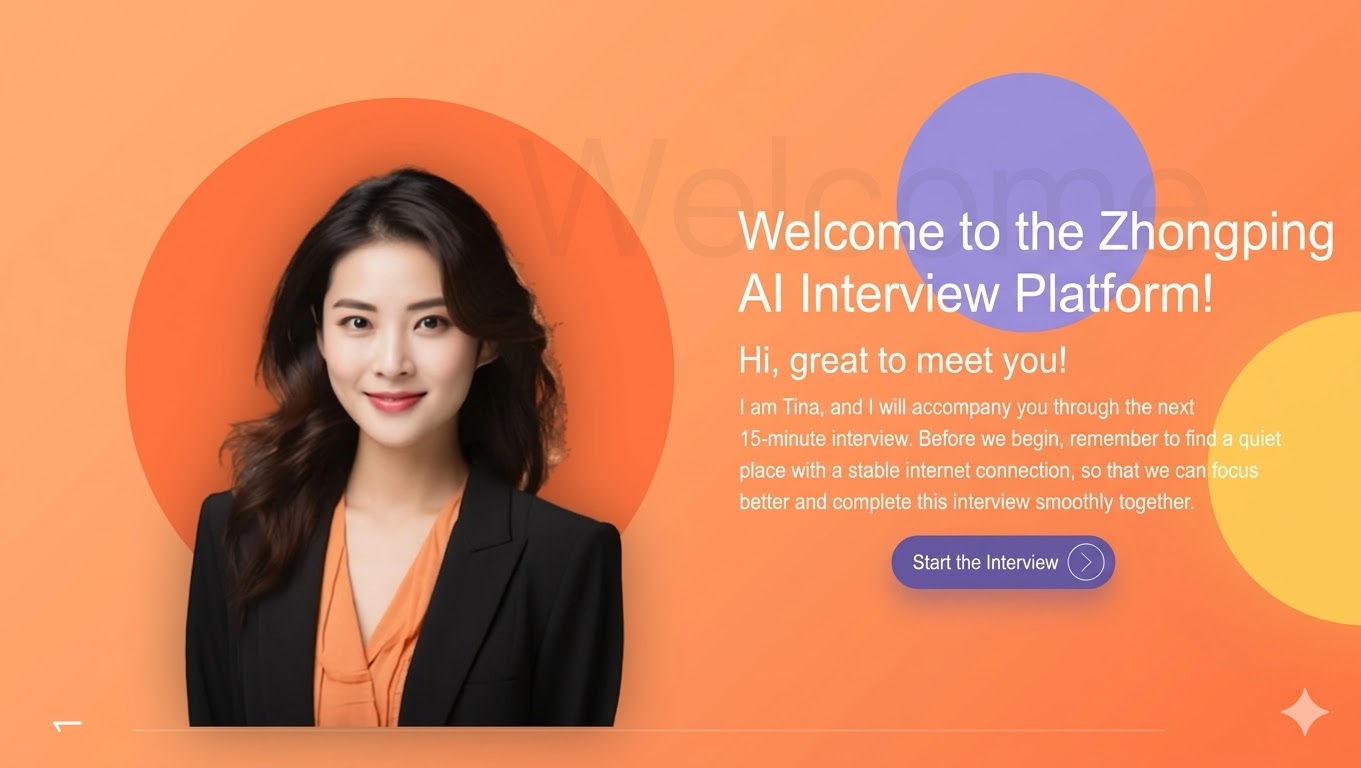
**

**Figure B1.** AI Interviewer Interface Presented to Participants (Human-Likeness Cue)


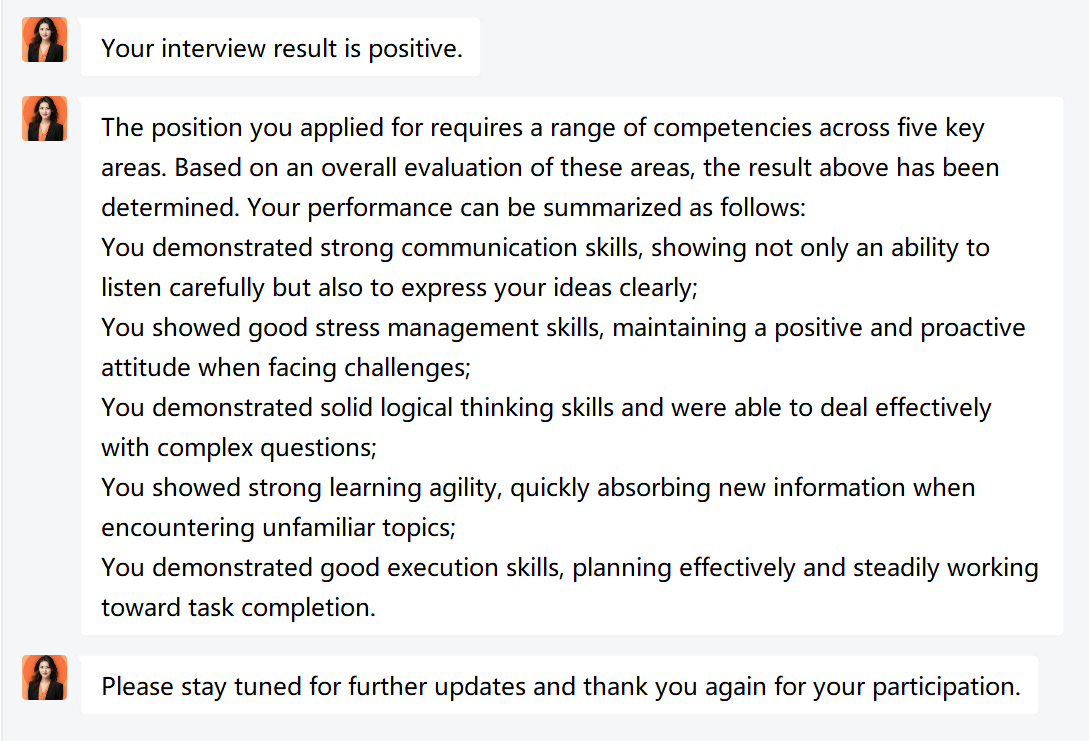


**Figure B2.** AI Interview Evaluation Feedback Example (Feedback Informativeness)


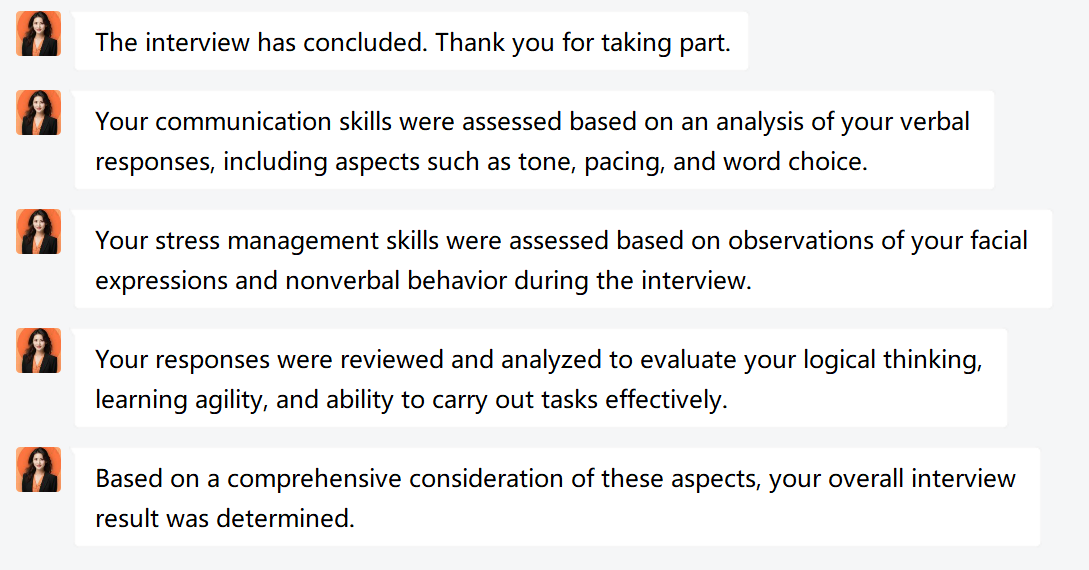


**Figure B3.** Explanation of AI Evaluation Criteria in the Interview System (Decision Transparency)
